# A Transcriptional Program Mediating Entry into Cellular Quiescence

**DOI:** 10.1371/journal.pgen.0030091

**Published:** 2007-06-08

**Authors:** Helen Liu, Adam S Adler, Eran Segal, Howard Y Chang

**Affiliations:** 1 Program in Epithelial Biology, Stanford University School of Medicine, Stanford, California, United States of America; 2 Department of Computer Science and Applied Mathematics, The Weizmann Institute of Science, Rehovot, Israel; North Carolina State University, United States of America

## Abstract

The balance of quiescence and cell division is critical for tissue homeostasis and organismal health. Serum stimulation of fibroblasts is well studied as a classic model of entry into the cell division cycle, but the induction of cellular quiescence, such as by serum deprivation (SD), is much less understood. Here we show that SS and SD activate distinct early transcriptional responses genome-wide that converge on a late symmetric transcriptional program. Several serum deprivation early response genes (SDERGs), including the putative tumor suppressor genes *SALL2* and *MXI1,* are required for cessation of DNA synthesis in response to SD and induction of additional SD genes. SDERGs are coordinately repressed in many types of human cancers compared to their normal counterparts, and repression of SDERGs predicts increased risk of cancer progression and death in human breast cancers. These results identify a gene expression program uniquely responsive to loss of growth factor signaling; members of SDERGs may constitute novel growth inhibitors that prevent cancer.

## Author Summary

Cells constantly sense their environment to decide whether to divide. Many genes that control the entry into cell division are known, and their excessive activation may cause cancer. In contrast, the way that cells cease to divide was thought to be a passive process, where signals for cell division gradually decay. In this study, the authors show that the decision to cease cell division and enter a state termed quiescence is also an active process. By monitoring the changes in activity over all genes, the authors identify a set of genes that respond specifically to decrements of external stimuli and ensure cessation of cell division. These genes act as brakes to prevent excessive cell division, and their inactivity is characteristic in many human cancers, particular those that progress to life threatening disease.

## Introduction

Quiescence, also termed G0, is defined as reversible cell cycle arrest where cells are poised to re-enter the cell cycle. Most eukaryotic cells spend the majority of their lifespan in the state of quiescence. In response to injury or specific extracellular stimuli, many types of somatic cells can quickly leave the quiescent state and enter the cell division cycle. For instance, in the skin, dermal fibroblasts and hair follicle stem cells are for the most part quiescent [1,2]. Injury to the skin stimulates fibroblasts and epidermal stem cells to rapidly proliferate; once tissue repair has been accomplished, the cells exit the cell cycle and reenter quiescence. Similarly, memory lymphocytes are quiescent as they circulate and survey the body, dividing only when stimulated by cognate antigenic stimuli to mount an immune response [3]. In addition to the absence of cell division, quiescent cells exhibit systematic differences in their metabolism and propensity for differentiation, which may help to ensure the reversibility of quiescence [4]. The ubiquity of quiescence as a central feature of cell life suggests that its regulation may be critical to normal development, degenerative diseases, and cancer [1,3,4].

Serum, the soluble fraction of clotted blood, is an important mitogenic signal in wound healing and tissue homeostasis. Many key genes involved in cell cycle entry were initially identified by their unique temporal patterns of expression in response to serum stimulation (SS) and are dysregulated in cancer [5]. In addition to cell cycle entry, serum induces a transcriptional program activating many aspects of wound healing [6]. This wound response program is recapitulated in many human cancers and is a strong predictor of tumor progression for these cancers [7,8]. While much is known about the signal transduction pathways, transcription factors, and immediate early genes that mediate the exit from quiescence and entry to the cell division cycle [5,6,9,10], comparatively much less is known about the mechanisms by which cells enter the quiescent state. Growth factor deprivation, contact inhibition, and loss of adhesion can each induce a shared set of quiescence genes [4], indicating the potential existence of multiple pathways to quiescence. Several tumor suppressor genes, such as *Rb* and *PTEN,* are required for quiescence maintenance in low serum conditions [11–13]. Yamamoto and colleagues have identified a set of antiproliferative genes whose repression requires ongoing activity of the mitogen-responsive kinase ERK during cell cycle progression [10]. The induction of these genes during quiescence is therefore simply a consequence of the absence of mitogen-induced signaling. If this mode of balanced regulation were generally applicable, then one might predict a symmetric network of gene regulation during quiescence entry and exit. Alternatively, an inducer of quiescence may engage a unique transcriptional program that is not regulated by cell cycle entry. Such quiescence entry-specific genes may represent novel growth inhibitors that link extracellular stimuli to the physiologic state of quiescence.

In this report, we characterize the genomic expression program of serum deprivation (SD) in fibroblasts and identify the predominance of asymmetric regulation in quiescence induction. We identify two putative tumor suppressor genes, *SALL2* and *MXI1,* as key regulators of the serum deprivation early response genes (SDERGs) and demonstrate the roles of the SDERGs in cell cycle exit and human cancer progression.

## Results

### Genomic Views of SS and SD Reveal That Early Response Genes Are Asymmetrically Regulated

To understand the genomic program of entry into quiescence, we characterized the temporal pattern of the genome-wide transcriptional profiles of fibroblasts in response to SD. We employed the same diploid fibroblast culture and experimental time points that we previously used to delineate a detailed transcriptional response to SS [7], thereby enabling a systematic comparison of entry and exit of quiescence. Human foreskin fibroblasts were grown in media containing 10% fetal bovine serum (FBS) for 48 hours, and following switch to low serum media containing 0.1% FBS, harvested at 15 time points ranging from 15 minutes to 48 hours after SD. Total RNA was extracted, amplified, and hybridized along with human universal reference RNA onto human cDNA microarrays containing ~43,000 elements, representing ~23,000 unique genes.

**Figure 1:**
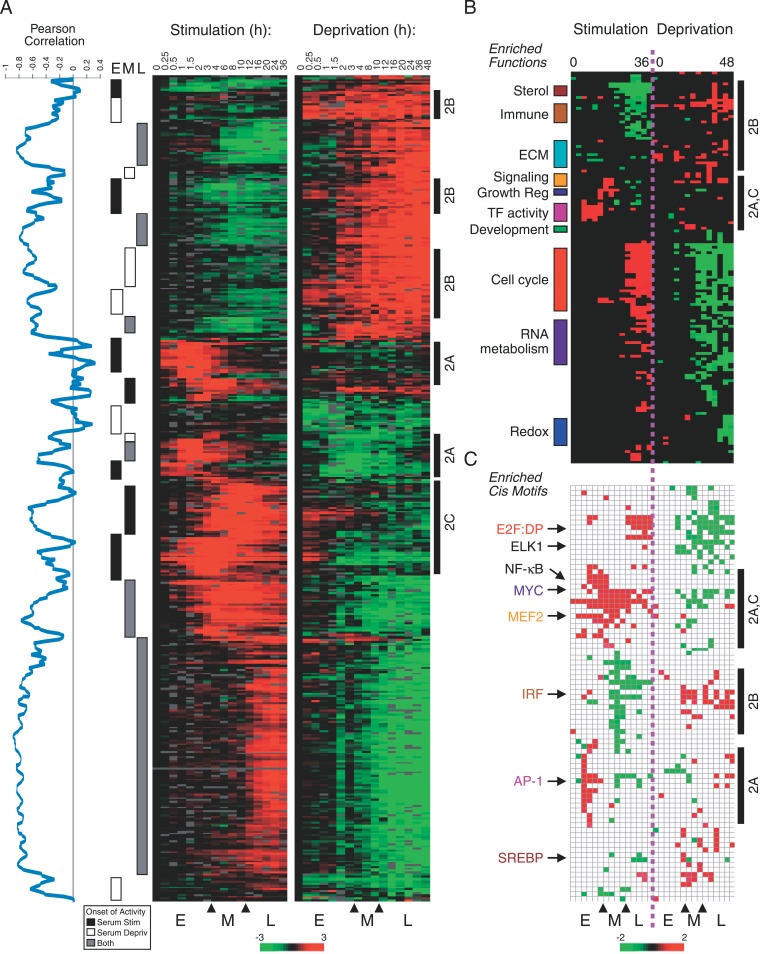
Systems Architecture of SS and SD in Human Fibroblasts (A) Global transcriptional response of fibroblasts to SS and SD is presented. On the right a heat map of gene expression profiles is shown. Each column is a time point; each row is a gene. Gene induction or repression relative to the zero time point of each time course is shown by the red-green intensity according to the bottom scale. Gene clusters that are examined in Figure 2 are marked by black bars to the right. In the middle a temporal pattern of gene regulation is shown. For each gene cluster, the onset of gene induction or repression in the SS or SD time courses is indicated by E, M, or L. Note the strong asymmetry of regulation in the early (E) (<3 h) time points, which gradually converge in the middle (M) (3–8 h) time points, and demonstrate symmetric regulation in the late (L) (>8 h) time points. On the left the Pearson correlation of gene expression profile in SS versus that of SD is presented. The average correlation value of ten nearest genes as grouped by hierarchical clustering is shown. (B) Module map of enriched gene ontology functions in the transcriptional programs of SS and SD is presented. Each column is a time point; each row is an enriched gene ontology term (*p* < 0.05; FDR < 0.05). For each gene ontology term, the average expression of the enriched member genes is shown by color intensity according to the scale bar. Select themes of gene ontology functions are highlighted on the left. (C) Module map of enriched *cis*-regulatory motifs in the transcriptional programs of SS and SD is presented. Functional groups and *cis* motifs based on the same underlying sets of genes are indicated by matching colors of solid bars and text on the left of (B) and (C), respectively.

Comparison of the temporal expression profiles of SD and SS revealed two dynamic programs with marked asymmetry in the early responses (Figure 1A). For each gene, we quantified the similarity of its pattern of expression during SD and SS by a Pearson correlation. (A correlation of −1 indicates exact opposite pattern; a correlation of 0 indicates no relationship; a correlation of +1 indicates identical pattern.) Strikingly, genes with an induction or repression onset after eight hours of treatment showed symmetric regulation by SS and SD with a Pearson correlation of −0.80 (i.e., genes induced by SS are repressed by SD and vice versa). In contrast, genes regulated within the first three hours of SS or SD were asymmetrically governed by these two stimuli (Pearson correlation −0.2 to +0.2). Genes that were regulated within three to eight hours of the treatments had an intermediate level of symmetry. These results thus suggest an asymmetric regulation of quiescence entry and exit with distinct sets of early response genes.

**Figure 2:**
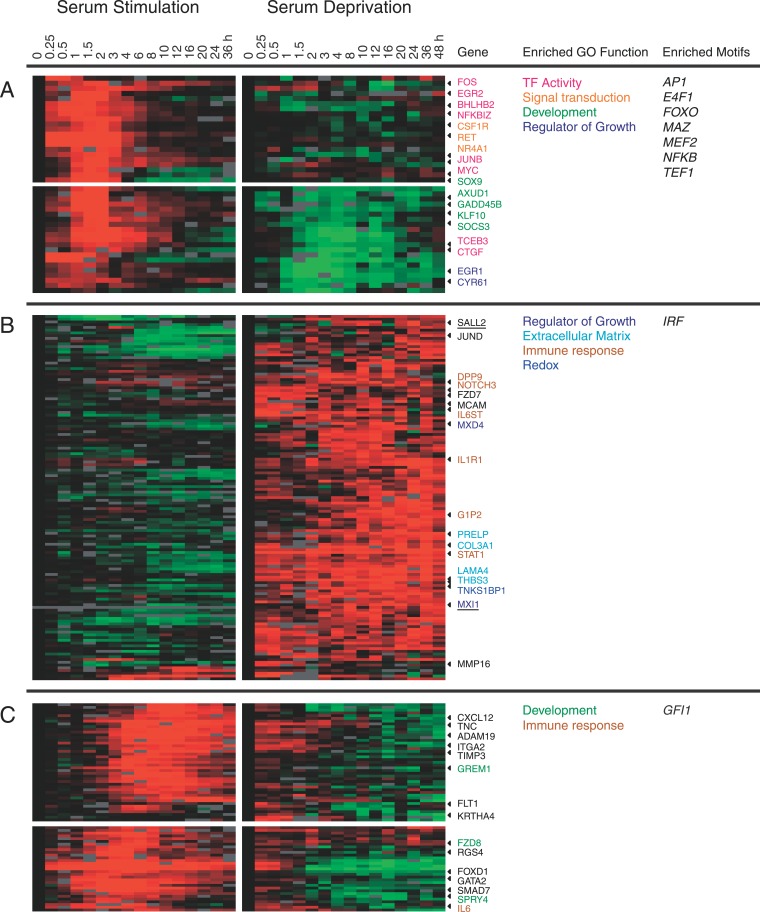
Early Response Genes Are Asymmetrically Regulated by SS and SD The transcriptional pattern, enriched gene ontology functions, and enriched *cis* motifs of early response genes to SS and SD. (A) Early response genes showing induction in SS are unresponsive or weakly responsive in SD. (B) Most early response genes showing induction in SD are not regulated in SS. (C) Some early response genes in SD show immediate induction followed by repression. The genes in (B) and (C) are collectively defined as the SDERGs.

To gain a higher order view of the transcriptional programs of SS and SD, we next identified the functional groups and upstream *cis*-regulatory sequences of genes that are regulated in expression by these two stimuli (Figure 1B and 1C). Using the module map method [14], we identified for each array the coordinate induction or repression of 1,735 gene sets, each defined as a group of genes encoding proteins possessing a shared biological function, biochemical process, or subcellular localization by gene ontology (*p* < 0.05; false discovery rate [FDR] < 0.05) (Figure 1B) [15]. This higher order view confirmed that many functions known to be regulated by serum [6], such as cell proliferation, RNA metabolism, and sterol synthesis, are symmetrically regulated by SS and SD in the late phase of each program (Figure 1B; Figure S1). In contrast, while coordinate induction of genes encoding transcription factors and signaling proteins characterized the early response of SS, the genes that were regulated in the early response of SD were enriched for functions in immune response, redox, and extracellular matrix metabolism (Figure 1B). The paucity of gene ontology terms describing functional groups characterizing early SD may reflect the current scarcity of knowledge about this cellular state.

Transcriptional regulation is mediated in large part by binding of *trans-*acting factors to *cis*-regulatory elements upstream of genes. To better understand the regulation of SS and SD, we therefore mapped the genome-wide occurrence of 175 phylogenetically conserved *cis*-regulatory motifs [16] in four kilobases surrounding transcriptional start sites genome-wide. For each array, we identified *cis*-regulatory motifs that are significantly enriched in the genes that are induced or repressed, yielding a map of the regulatory motifs active in quiescence entry and exit (Figure 1C). This unbiased approach highlighted many known transcription factors that play key roles in this process and again reveals the marked asymmetry in the early response of SS and SD (Figure 1C). For instance, the gene set defined by enrichment of motifs for E2F and DP transcription factors contained many cell cycle genes and was symmetrically regulated late in the response by SS and SD (Figure 1C; Figure S1). In contrast, gene sets defined by the motif of AP-1, MEF2, or NF-κB were induced early in response to SS but were not substantially regulated by SD. Once again, we noted a relative paucity of *cis* motifs that identify early response genes to SD. This result may reflect incomplete information of the relevant transcription factors and their cognate *cis* motifs or may reflect additional post-transcriptional mechanisms in regulating mRNA levels in SD.

### SD Activates a Unique Gene Expression Program to Induce Cellular Quiescence

To begin to understand the asymmetric regulation of quiescence entry and exit, we focused on genes that are induced early in response to either SS or SD. Approximately half of the well-known early response genes to SS, such as *EGR1, CYR61,* and *GADD45B,* were correspondingly repressed by SD, albeit with lower amplitude and delayed kinetics that peak at approximately three hours after SD (Figure 2A). In contrast, other key SS early response genes, such as *FOS, JUNB,* and *MYC,* are transiently induced by SS and showed little change in transcript level in SD (Figure 2A). Conversely, a group of 135 genes defined by early induction in response to SD, which we term SDERGs, showed immediate induction within the first three hours after SD, but demonstrated substantially less or delayed regulation by SS (Figure 2B and 2C; Table S1). Some SDERGs, including *SALL2, MXI1,* and *TNKSBP1,* are induced in a sustained manner by SD while other SDERGs, such as *SPRY4* and *SMAD7,* are induced by SD in a transient manner. Intriguingly, *SALL2* and *MXI1* are both putative tumor suppressors that can suppress cell growth when overexpressed [17,18]. *SALL2* encodes a zinc finger transcription factor and is a homolog of *Drosophila* homeotic gene *Spalt*. Human *SALL2* resides in a chromosomal region frequently deleted in ovarian cancers, and SALL2 protein is a binding target of the oncogenic large T-antigen from polyoma virus [19]. MXI1 is a member of the MAD family of potent antagonists of MYC oncoproteins [20]. MXI1 resides on a locus in human Chromosome 10 that is deleted in several types of human cancers, including prostate cancer, and deletion of MXI1 in mouse leads to a cancer-prone phenotype [18]. We noted that several SDERGs are well-known interferon-induced genes, such as *STAT1, ILIR1, BDKRB2,* and *PLSCR1*; several additional SDERGs, such as *JUND, IFIT2,* and *G1P2* are predicted by our *cis*-regulatory map to contain a motif for the interferon regulatory factor (IRF) family of transcription factors. A likely candidate is IRF1 because IRF1 can induce several of these genes, is itself induced by SD, and possesses antiproliferative properties [21,22]. The asymmetric regulation of early response genes suggests that the transition to a quiescent state may be enforced by employing signaling pathways unique to SD.

### *SALL2* and *MXI1* Are for Cell Cycle Exit and Gene Expression in Response to SD

**Figure 3:**
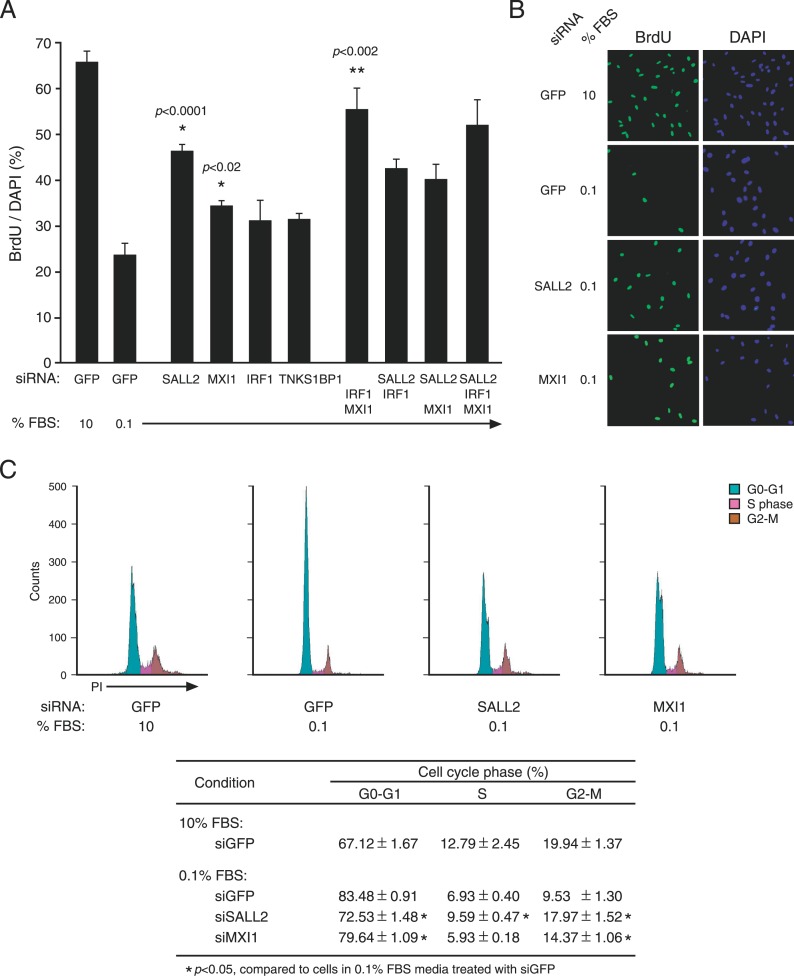
Two SDERGs, SALL2 and MXI1, Are Required for Cell Cycle Exit in Response to SD (A) SALL2 and MXI1 are required for cell cycle exit. Percentage of BrdU-positive cells with SD and with treatment of the indicated siRNAs are shown (mean ± standard error). *, *p* < 0.05 compared to the effect of siGFP by Student's t-test. **, *p* < 0.05 compared to the strongest effect of the single gene knockdown. (B) Immunofluorescence of BrdU incorporation for cells treated with siGFP, siSALL2, and siMXI1 is shown. (C) The upper panel shows cell cycle profiles of cells with the indicated treatments as determined by FACS analysis. PI, propidium iodide staining. The lower panel shows quantification of four replicates (average ± standard error) of the indicated cell cycle stages.

To address the functional role of the SDERGs, we used RNA interference to examine the requirement of specific SDERGs for cell cycle exit. We selected four candidate genes *(SALL2, MXI1, IRF1,* and *TNKS1BP1)* that encode transcription factors or signaling proteins that may regulate quiescence induction. TNKS1BP1, a putative telomere binding protein, was included because of the reported roles of telomerase in enhancing S-phase progression [23,24]. We transfected primary human foreskin fibroblasts grown in high serum (10% FBS) media with silent interfering RNA (siRNA) pools corresponding to each of four candidate genes or a control siRNA targeting *GFP*. The cells were switched to 0.1% serum media 72 hours after transfection to induce quiescence, and DNA synthesis was measured 16 hours later by 5-bromo-2′-deoxyuridine (BrdU) incorporation. Reverse transcription-PCR confirmed decreased expression of the target mRNAs (Figure S2). In control cells treated with siRNAs targeting GFP, SD lowered the percentage of BrdU+ cells from 65% to approximately 25%, indicating induction of quiescence and efficient cell cycle exit (Figure 3A and 3B). Strikingly, cells treated with siRNAs targeting *SALL2* doubled the number of BrdU+ cells after SD (*p* < 0.0001), while siRNAs targeting *MXI1* showed modest but consistent increase in BrdU+ cells (*p* < 0.02). siRNAs to *IRF1* or *TNKS1BP1* did not significantly affect quiescence induction under the conditions tested. To test the potential functional relationships between SALL2, MXI1, and IRF1, we treated cells with pair-wise combinations of siRNAs. Silencing of IRF1 strongly cooperated with silencing of MXI1, but silencing of neither IRF1 or MXI1 cooperated with silencing of SALL2 to prevent cell cycle exit. Fluorescence-activated cell sorting analysis of DNA content confirmed that depletion of SALL2 or MXI1 blocked the ability of cells to arrest in G0–G1 after growth factor deprivation and instead led to inappropriate progression through S and G2/M phases of the cell cycle (Figure 3C). These results suggest that several of the SDERGs identified by our microarray screen are required for entry to cellular quiescence and that SALL2 and MXI1 may trigger different pathways to enforce quiescence.

To further delineate the mechanisms of SALL2 and MXI1 action, we identified genes that required SALL2 and MXI1 for proper regulation during quiescence induction. RNA from cells transfected with silent interfering (si)SALL2 and siMXI1 was extracted, amplified, and compared with RNA from cells treated with siGFP on cDNA microarrays.

**Figure 4:**
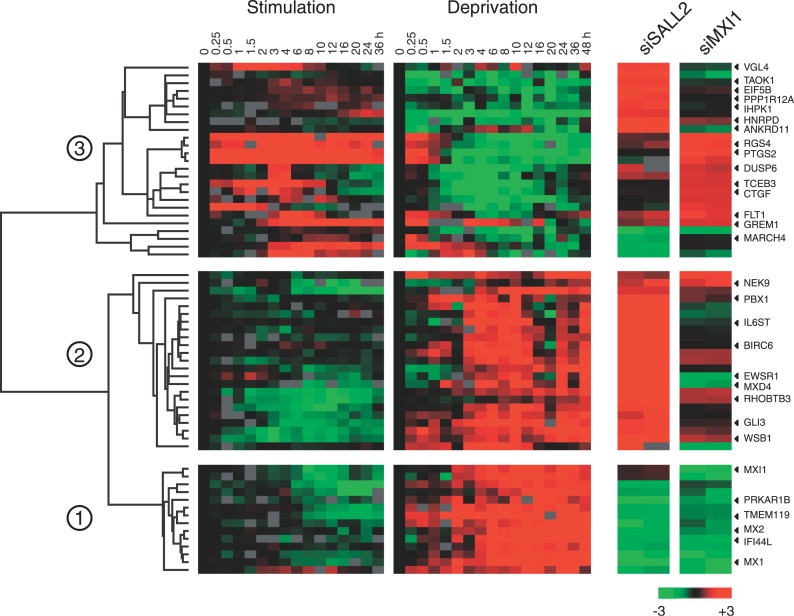
SALL2 and MXI1 Are Differentially Required for Gene Regulation in SD On the right, altered global transcriptional programs are shown 16 h after SD by transfection with siSALL2 or siMXI1, each compared against siGFP reference RNA and performed in duplicates. Red indicates higher expression in cells treated with siSALL2 or siMXI1 compared to cells treated with siGFP; green indicates the lower expression in siSALL2 or siMXI1 samples versus siGFP. On the left, the temporal expression program of the same genes SS and SD is shown. The genes are grouped by their similarity of expression by hierarchical clustering; the three clusters indicated on the far left are as described in the text.

Genes whose expression levels were consistently changed by loss of SALL2 or MXI1 were identified, and their temporal regulation by SS and SD were systematically organized by hierarchical clustering. We observed three main patterns of gene regulation. First, both SALL2 and MXI1 are individually required for the induction of a cluster of SD middle response genes (Figure 4, cluster 1). After knockdown of either SALL2 or MXI1 in SD, these genes reverted to a pattern of expression more closely resembling their normal behavior in SS than in SD. Second, in contrast to this shared role in SD gene induction, SALL2 and MXI1 acted to repress mutually exclusive sets of SD-repressed genes (Figure 4, cluster 3). Third, SALL2 appears to have a unique role in limiting expression of a set of middle response genes to SD, as their expression became super-induced when SALL2 was silenced (Figure 4, cluster 2). These results confirm the distinct roles of SALL2 and MXI1 in quiescence induction and suggest multiple roles for SALL2 in gene regulation throughout quiescence entry and maintenance.

### Widespread Repression of SDERGs in Human Cancers

**Figure 5:**
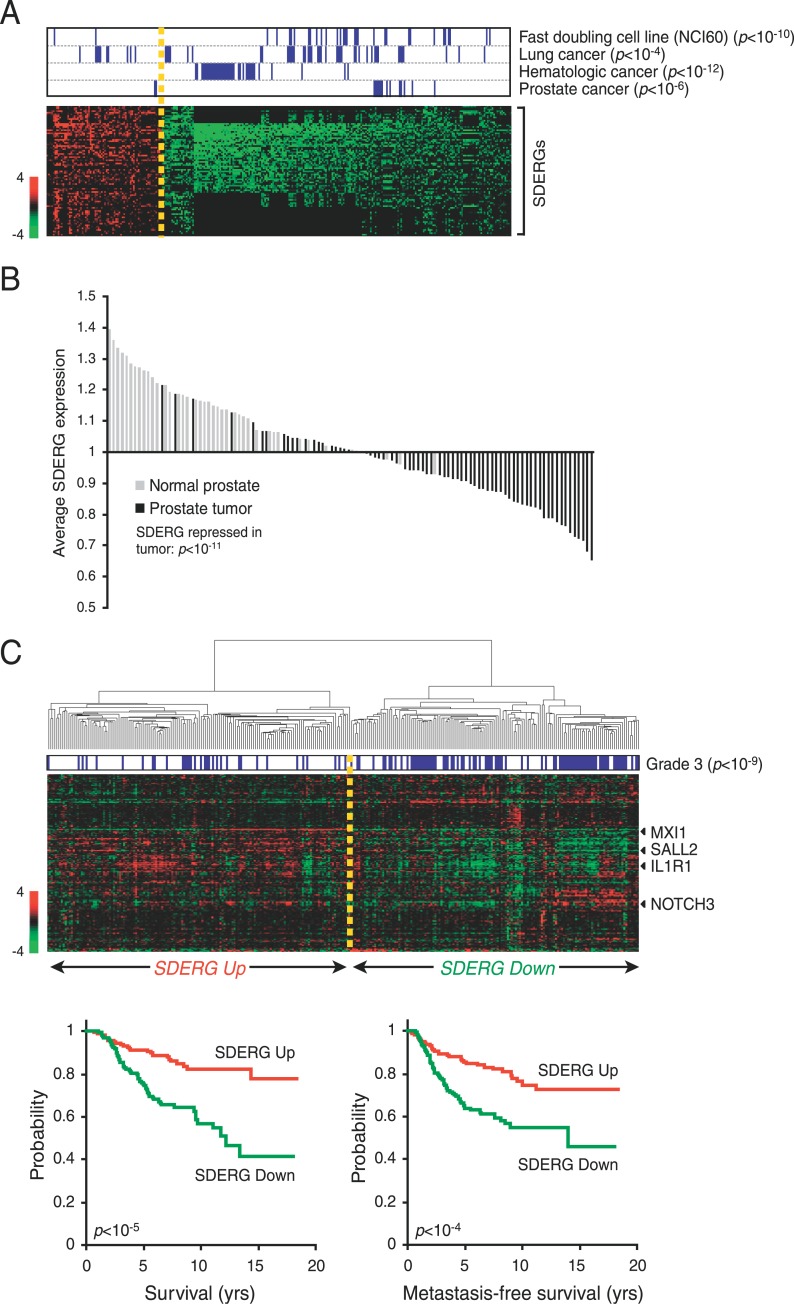
SDERGs Are Coordinately Repressed in Human Cancers (A) SDERGs are coordinately repressed in highly proliferative cells and human cancers. We interrogated a compendium of 1,973 microarrays representing 22 human tumor types and diverse normal controls for coordinate regulation of the SDERGs. In the heat map displayed, each column is a sample showing coordinate induction or repression of SDERGs (*p* < 0.05, FDR < 0.05, hypergeometric distribution); each row is a SDERG. In the top matrix enriched clinical annotations of microarrays show SDERG repression. Each microarray in the compendium was annotated with biological and clinical information of the sample; we therefore queried each annotation for enrichment of SDERG induction or repression. Each blue hatchmark indicates a sample with the indicated annotation. Each row is an enriched annotation (*p* < 0.05, FDR < 0.05). A total of four examples in the top ten enrichments are shown. (B) Repression of SDERGs distinguish prostate cancer from normal prostate in an independent dataset of 103 samples [25] (*p <* 10^−11^, chi-square test). (C) Repression of SDERGs in human breast cancer predicts increased metastasis and poor survival. In the upper panel hierarchical clustering with SDERGs separate 295 stage I and II early breast tumors into two groups, with predominant induction or repression of SDERGs. In the lower panel Kaplan-Meier survival curves show worse survival (*p <* 10^−5^) and increased metastasis (*p <* 10^−4^) of patients with tumors that repressed SDERGs.

Having discovered the SDERGs as a set of 135 genes specifically induced when fibroblasts enter quiescence, we next tested whether the SDERGs might have broad roles in growth inhibition. We reasoned that if SDERGs were generally required to induce cell quiescence, then SDERGs might be coordinately repressed in conditions of excessive cell proliferation, such as in cancer. We therefore interrogated a compendium of 1,973 microarrays representing 22 human tumor types to search for enriched coregulation of the 135 SDERGs, using the gene module map method [14]. The SDERGs were indeed coordinately repressed in many conditions that represent pathologic proliferation, specifically the subset of fast doubling cell lines in the NCI60 collection of tumor cell lines (*p* < 10^−10^) and several human cancers relative to their normal tissue counterpart including cancers of prostate (*p* < 10^−6^), blood (*p* < 10^−12^), and lung (*p* < 10^−4^) (Figure 5A). These results suggest that SDERGs likely antagonize cell proliferation in many cell types.

Cancer consists of a broad range of clinical behaviors ranging from indolent tumors to aggressive metastatic disease. To further dissect the potential molecular variation underlying this clinical heterogeneity and to extend and validate our results, we tested the prognostic power of the SDERG gene set in independent datasets and different subtypes of human cancer. Analyzing DNA microarray data from a study of 103 prostate tissues and cancer [25], we found that coordinate repression of SDERGs could identify over 90% of prostate tumors relative to normal prostate, a finding very unlikely due to chance alone (*p* < 10^−11^) (Figure 5B). Furthermore, expression of SDERGs in a set of 295 breast cancers [26] naturally divided the breast tumors into two groups (Figure 5C). Patients with breast cancers that diminished expression of SDERGs had significantly worse survival (*p* < 10^−5^) and significantly increased probability of metastasis (*p* < 10^−4^). This group of tumors with repression of SDERGs also tended to be of the grade 3 tumors (*p* < 10^−9^), which are defined by higher cell proliferation and less differentiation. Interestingly, the mRNA levels of SALL2 and MXI1, either alone or in combination, were insufficient to predict overall survival or metastasis-free survival (*p* > 0.05, Cox-Mantel test); conversely, removal of SALL2 and MXI1 from SDERGs did not affect the prognostic power of the SDERG gene set (unpublished data). These results further suggest a role for SDERGs to prevent excessive and pathologic proliferation. By reflecting the propensity for quiescence, the expression level of the SDERG gene set as whole may aid in predicting tumor behavior in two of the most common human cancers.

### Quiescence Initiation versus Quiescence Maintenance

**Figure 6:**
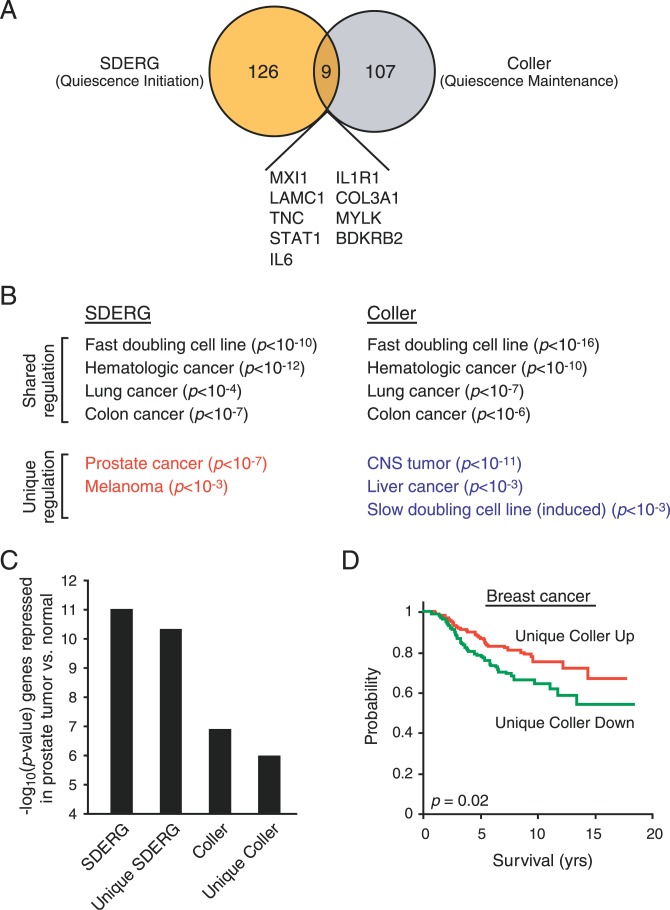
Comparison of SDERGs with Genes Associated with Quiescence Maintenance (A) Overlap of SDERGs with 116 quiescence maintenance genes defined by Coller et al. [4] is shown. (B) Comparison of coordinate regulation of SDERG or 116 quiescence maintenance genes in human cancers is presented. Shown are select enriched clinical annotations in the subset of 1,973 microarrays representing 22 human tumor types where the gene set of interest is coordinately repressed (*p* < 0.05; FDR < 0.05). One exception is the induction of quiescence maintenance genes in the slow doubling subset of the NCI60 cell lines. (C) Repression of SDERG versus quiescence maintenance genes in prostate cancer relative to normal prostate tissue is presented. Shown is the negative log of the *p* value of the concordance of gene expression compared to that expected from chance alone (chi-square test). (D) Modest prognostic power of the 107 unique quiescence maintenance genes for predicting overall survival in primary human breast cancers is presented.

To better understand the transcriptional regulation of quiescence initiation versus maintenance, we compared SDERGs (135 genes) with 116 genes previously found to be concordantly induced by prolonged entry into quiescence, four days after growth factor deprivation, contact inhibition, or loss of adhesion [4]. A total of nine genes were in common between these two gene sets, while only one overlap gene is expected by chance alone (*p* < 10^−7^, hypergeometric distribution). The overlap genes include *MXI1* and *STAT1,* thus indicating an interesting but limited overlap between the SDERGs and genes expressed during quiescence maintenance (Figure 6A). We next examined the coordinate expression of these 116 quiescence maintenance genes in 1,973 microarray representing 22 human tumor types (Figure 6B). SDERGs and quiescence maintenance genes showed overlapping but distinct patterns of expression, with some tumors coordinately repressing both gene sets but many that repress only one of the two sets. In human prostate cancer, quiescence maintenance genes are typically repressed but in far more haphazard fashion compared to SDERGs (*p* value of the separation is five logs of magnitude worse) (Figure 6C). Similarly, coordinate repression of quiescence maintenance genes is modestly predictive of primary breast cancer survival but not predictive of metastasis-free survival (Figure 6D and unpublished data). These results suggest that genes mediating entry into quiescence are largely distinct from those associated with quiescence maintenance, and the two programs may be repressed in distinct fashions to facilitate the progression of specific types of human cancers.

### SDERG Induction Is Distinct from Stress Response

**Figure 7:**
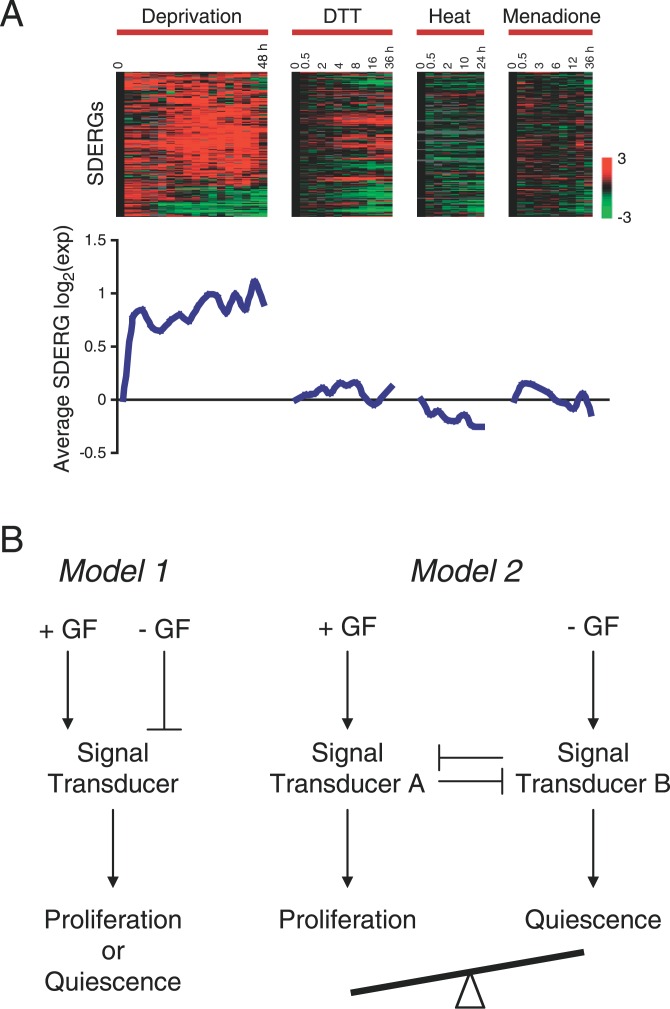
SDERGs Are Unique Responders to Growth Factor Deprivation (A) SDERGs are not induced by diverse cell stress. Comparison of heat map and average expression levels of SDERGS in response to SD, dithiothreitol, heat, and methadione is presented. (B) Models of entry into cell quiescence are presented. In Model 1, cells interpret gain and loss of growth factor signaling by one set of common genes, leading to a decision to engage proliferation or quiescence. Quiescence can be just the absence of positive signals for proliferation. In contrast, Model 2 incorporates the discovery of SDERGs as a transcriptional program uniquely responsive to loss of growth factor signaling and mediates quiescence entry. Two distinct sets of early response genes actively signal the gain and loss of growth factor signaling, the balance of these two transcriptional programs determines proliferation versus quiescence.

The general association between SDERGs and cell cycle exit raises the possibility that SDERGs may be induced by additional stimuli. For instance, in response to variety of noxious stress, cells will exit the cell cycle as part of the stress response. To test the possibility that SDERGs may be induced by stress, we queried the expression pattern of SDERGs in the published gene expression data of fibroblasts exposed to multiple types of stress [27]. In contrast to the strong induction of SDERGs by SD, exposure of fibroblasts to the reducing agent dithiothreitol (causing protein unfolding and endoplasmic reticulum stress), heat shock, or menadione (inducing oxidative stress) did not induce the SDERGs (Figure 7A). These results reaffirm SDERGs as a program uniquely responsive to loss of growth factor signaling as represented by SD.

## Discussion

### Asymmetric Regulation of Quiescence Entry and Exit

By determining the genomic transcriptional program in response to SS and SD, we observed the asymmetric regulation of quiescence entry and exit (Figure 1). While the late transcriptional responses to these two opposing stimuli are largely symmetric, this symmetric program results from two distinct early transcriptional responses. These data suggest that in addition to previously identified antiproliferative genes that require ongoing growth factor signaling for their suppression [10], a major mechanism of quiescence entry is the induction of a set of unique quiescence entry genes (Figure 2). By simple analogy, a speeding car may be slowed by releasing the gas pedal, but the car can also be brought to a screeching halt by releasing the gas pedal and stepping on the brakes. We suggest that the SDERGs may be a set of brakes for cell cycle progression and growth factor-induced gene expression. Just as a gain of growth factor signaling activates the classic immediate early genes to induce cell cycle entry, a loss of growth factor signaling uniquely activates the SDERGs to induce cell quiescence. The decision of cell proliferation or quiescence is thus determined by the balance of growth factor-induced genes and SDERGs (Figure 7B). SDERGs are also actively involved in the repression of growth factor-induced genes. For instance, SALL2 and MXI1 are required to repress distinct sets of serum-inducible genes (Figure 3C); in the case of MXI1 this may occur by direct competition with MYC for binding to promoters of serum-inducible genes [20]. Thus, there is likely cross regulation of the early transcriptional responses to growth factor stimulation and deprivation.

One potential reason for this dual transcriptional response to growth factor gain and loss is to achieve tight regulation. It has been well known that many early response genes to SS are induced as a precise pulse that then rapidly decays despite continued mitogen presence (Figure 2A), thereby providing a check against unlimited proliferation and the risk of cancer. For instance, the classic mitogen-induced proto-oncogene *MYC* is regulated by transcriptional autorepression [28], mRNA instability [29], and rapid protein turnover [30,31]; enforced *MYC* expression is sufficient to induce ectopic DNA replication and DNA damage within just one cell cycle [32]. The low level of *MYC* and other growth factor early response genes at steady state would render a system based on their further decrement an insensitive strategy to detect growth factor deprivation. Instead, a decrement of growth factor signaling triggers a robust transcriptional response of SDERGs, leading to additional quiescence gene induction and exit of cell cycle. Intriguingly, a large number of SDERGs (including *SALL2* and *MXI1,* which are required for entry to quiescence) are induced and maintained in stable expression in response to SD (Figure 2B). The longevity of SDERG expression in contrast to the transient expression of growth factor early response genes may provide an explanation for cell quiescence as the default state of most eukaryotic cells.

### *SALL2* as a Mediator of Cell Quiescence

Among the several the SDERGs we tested, silencing of *SALL2* expression had the most substantial effect on cell cycle exit in response to growth factor deprivation. Previously, Benjamin and colleagues had identified *SALL2* as an antiproliferative gene by virtue of a tumor host range selection procedure for the oncogenic polyoma virus [19]. Enforced *SALL2* expression in ovarian cancer cells inhibits tumor xenograft growth in vivo and can induce the expression of cyclin-dependent kinase inhibitor p21, although it is unclear whether p21 is the sole mechanism by which *SALL2* elicits cell cycle arrest [17]. The biological context in which *SALL2* might exert its antiproliferative effect was also not known. Our results suggest that SALL2 is induced in response to loss of growth factor signaling (Figure 2B). Acute loss of SALL2 during SD blocked the ability to stop DNA synthesis and induce additional quiescence-associated genes, suggesting that SALL2 is required for quiescence induction in response to growth factor deprivation (Figure 3C). Among the mammalian SALL family of zinc finger transcription factors, mutation of *SALL1* leads to developmental abnormalities [33], and *SALL4* is required for maintenance of pluripotency in embryonic stem cells [34,35]. Surprisingly, *SALL2* knockout mice are viable and have no obvious phenotype [36], raising the possibility that *SALL2*'s function may be redundant or compensated by other SALL family members. Indeed, we found that knockdown of *SALL4* also blocks cessation of DNA synthesis in response to SD even though S*ALL4* mRNA level does not change in response to SD (H. Liu and H. Y. Chang, unpublished data). Thus, it may be the total pool of SALL transcription factors in the cell that determines cell quiescence, and SALL4 may compensate for the chronic loss of SALL2 during development. Coller et al. have shown that quiescence in fibroblasts inhibits their *trans-*differentiation (such as into muscle cells in response to enforced MyoD expression) [4]; the role of SALL transcription factors in cell quiescence may therefore be intimately linked to their roles in stem cell pluripotency [34,35]. The mechanisms by which the *SALL2* message accumulates during SD and the functional roles of newly discovered SALL-regulated genes in SD should be addressed in future studies.

### Repression of SDERGs in Human Cancer

Because cell quiescence has been postulated to be an important safeguard against cancer [1], we reasoned that a transcriptional program mediating entry into quiescence might be systematically repressed in human cancers. Our survey of nearly 2,000 microarrays representing diverse types of human cancers identified multiple tumor types in which SDERGs are coordinately repressed (Figure 4). In addition, we found that repression of SDERGs unambiguously distinguished prostate cancers from adjacent prostate tissues, and repression of SDERGs in human breast cancers further predicted aggressive clinical course of early stage tumors. These properties are specific to SDERGs and are present to a much lower extent in genes associated with quiescence maintenance (Figure 6). Combined with the evidence that several SDERGs are required for cell cycle exit, these results highlight a potentially important role for the ability of cells to sense decrements of growth factor signaling and respond by quiescence. SDERGs and other genes induced by stimuli that induce cell quiescence may represent previously unrecognized tumor suppressor pathways; better understanding of these transcriptional programs may lead to new avenues of cancer diagnosis and treatment.

## Materials and Methods

### Cell and tissue culture.

Primary human foreskin fibroblasts (CRL 2091; American Type Culture Collection, http://www.atcc.org) were cultured in DMEM plus 10% FBS. Cells were plated at 10% confluence. Cells were switch to DMEM plus 0.1% FBS 48 h after the last passage and harvested at the indicated time points.

### Microarray procedures.

Construction of human cDNA microarrays containing approximately 43,000 elements, representing approximately 23,000 different genes, and array hybridizations were as previously described [8]. Total RNA was extracted using Trizol according to the manufacturer's instructions (Invitrogen, http://www.invitrogen.com) and amplified using the Ambion MessageAmpII aRNA kit (Ambion, http://www.ambion.com). For time course experiments, Human Universal Reference RNA (Stratagene, http://www.stratagene.com) was used as reference RNA to compare with RNA from individual time points. We took four independent samples at time zero, which functioned as the baseline for other sample time points. For siRNA samples, siGFP was used as the reference RNA to compare with RNA from cells transfected with siSALL2 or siMXI1; each comparison was performed in duplicate. Full microarray data are available for download at Stanford Microarray Database (http://genome-www5.stanford.edu) or Gene Expression Omnibus (http://www.ncbi.nlm.nih.gov/geo).

### Data analysis.

We selected genes for which the corresponding array elements had fluorescent hybridization signals at least 1.5-fold greater than the local background fluorescence in the reference channel and further selected genes for which technically adequate measurements were obtained from at least 80% of the samples in a given dataset. The zero time point for the SD experiment was performed in quadruplicate, and the four gene expression measurements were averaged and subtracted from those of the subsequent time points in order to visualize gene induction or repression over time. The gene expression profiles of the same cells to SS over 15 time points were similarly transformed by subtraction of expression value from each time point by that of the zero time point. The two datasets were then merged by matching Stanford clone IDs of the cDNA probes. We next focused on genes that exhibited substantial variation in expression and selected the subset of genes that were induced or repressed by at least four-fold in at least one array in either time course, yielding 444 cDNA probes (henceforth genes). These genes were organized and grouped based on the similarity in their patterns of expression by average linkage clustering using the Cluster software [37]. Clustering of genes revealed three main transition points of gene expression variation—immediately after SS or SD, three hours after the stimuli, or eight hours after the stimuli. We therefore defined a time for the induction or repression of each gene cluster as the time point at which the gene expression reaches half maximal induction or repression, and classified each cluster as being regulated early (<3 h), middle (3–8 h), and late (>8 h). For gene clusters that exhibit biphasic or more complex patterns of regulation, we defined the onset of activity based on the first peak of expression variation. To quantify the degree of divergence among early-, mid-, and late-response genes, we calculated the Pearson correlation between the expression pattern of the SS and SD time courses for each gene. After ordering genes by hierarchical clustering, we visualized the Pearson correlation of gene clusters by displaying the moving average of correlation values of the ten nearest genes along the y-axis of the heat map.

The gene module map of functional groups of genes was as described using the software package Genomica [14]. Briefly, for each microarray, we identified genes that were induced or repressed by at least 2-fold and tested for their enrichment in each of 1,735 gene sets defined by gene ontology terms [15] using the hypergeometric distribution. An FDR calculation was used to account for multiple hypothesis testing. Enrichments that had *p* < 0.05 and FDR < 0.05 were considered significant and are shown in Figure 1B, yielding a higher order view of each gene expression profile as sets of activated and repressed functions.

For the *cis-*regulatory motif map, we first defined motif modules from the data of Xie et al. [16], where each motif module is a group of genes that shared enrichment in an evolutionarily conserved *cis*-regulatory motif in their upstream regulatory sequences. The upstream regulatory region of each gene is defined as the 4,000 base pairs centered at the annotated transcription start site, as was done by Xie et al.; 175 motif modules were defined. Second, for each array, we identified genes that were induced or repressed by 2-fold and tested for their enrichment in each of the motif modules using the hypergeometric distribution as described above. Modules with significant enrichment (*p* ≤ 0.05) were identified and shown in Figure 1C, yielding a higher level view of each expression profile as a combination of activated and repressed *cis-*regulatory motifs.

To conduct microarray analysis of siRNA treated cells we employed a type I microarray design where mRNA of cells treated with siSALL2 or siMXI1 (labeled with Cy5) was compared to mRNA of cells treated with siGFP (labeled with Cy3) by competitive hybridization to cDNA microarrays. We selected for analysis genes for which the corresponding array elements had fluorescent hybridization signals at least 1.5-fold greater than the local background fluorescence in the reference channel, and we further restricted our analyses to genes for which technically adequate measurements were obtained from both duplicate arrays. We further selected genes that were induced or repressed by at least 2-fold in two arrays by siSALL2 or siMXI1, and the genes were organized by average linkage clustering.

Regarding the cancer compendium and clinical outcome data, a cancer compendium of 1,973 microarrays was as described [14]. Gene probes from different microarray platforms were mapped by LocusLink identification numbers ((http://www.ncbi.nlm.nih.gov/entrez/query.fcgi?db=gene). To test for the coordinate regulation of SDERGs in human cancers, we defined the 135 SDERGs as one gene set and tested each expression profile in the compendium for coordinate induction or repression of the SDERGs using the module map method in Genomica [14]. Specifically, for each microarray, we identified genes that were induced or repressed by at least 2-fold, and tested for their enrichment in SDERGs over that expected by chance alone (*p* < 0.05, FDR < 0.05). Next, to identify enriched clinical annotations among samples that exhibit coordinate SDERG induction or repression, for each annotation we compared the frequency of SDERG induction or repression among the samples versus that expected by chance alone. We found many significant enriched annotations of SDERG repression but not of SDERG induction in cancer (*p <* 0.05, FDR < 0.05, hypergeometric distribution). Several of the top ten enriched clinical annotations are reported in Figure 5A. The exact same procedure was repeated for 116 quiescence maintenance genes defined by Coller et al. [4].

To examine the expression of SDERGS in an independent set of human prostate cancer, we used the published prostate cancer microarray data of Lapointe et al. [25]. Gene probes were matched by cDNA clone IDs as this dataset was also generated on Stanford cDNA microarrays. We computed the average expression value of the 135 SDERGs in each of the 103 samples and rank ordered the average SDERG expression values. We considered those samples with average SDERG expression value greater than the mean of all 103 samples to have induction of SDERGs and those samples with average SDERG expression value to be below the mean of all 103 samples to have repression of SDERGs. The significance of the observed grouping of over 90 percent of prostate cancers with repression of SDERGs compared to normal prostate was evaluated by a two-by-two chi-square test, yielding a *p* value of 10^−11^. *p* Values were also calculated using the same procedure for the 116 quiescence maintenance genes and the 107 genes unique to Coller et al. [4] and plotted in Figure 6C as the −log_10_(*p* value).

To examine the clinical significance of SDERG repression in human breast cancer, we used the published breast cancer microarray data of van de Vijver et al. [26]. Gene probes were matched by Unigene ID (http://www.sgn.cornell.edu/bulk/input.pl?mode=unigene), and the 295 breast cancer samples were organized by two-way hierarchical clustering based on the expression pattern of the SDERGs. The main bifurcation of the dendrogram separated the breast cancer samples into two groups, one group with coordinate induction of SDERGs (termed “SDERG up”) and one group with coordinate repression of SDERGs (termed “SDERG down”). We compared the differences in overall survival and metastasis-free survival of these two groups of patients as defined by SDERGs using the Cox–Mantel test in the program Winstat (R. Fitch Software, http://www.winstat.com). Data from the 295 breast cancer samples were obtained for the quiescence maintenance genes unique to Coller et al. [4] and the same procedures as described above were performed.

To examine the expression pattern of SDERGs in fibroblasts undergoing cell stress, we downloaded the published microarray data of Murray et al. [27]. Gene probes were matched by Stanford cDNA clone IDs. Expression of SDERGs during SD or exposure to DTT, heat, or menadione are shown as heat maps, and the average expression values are shown across each time course as a graph in Figure 5A.

### RNA interference and cell cycle analysis.

Cells were transfected with 20 nM of siRNA pools corresponding to each of the target genes *(SALL2, MXI1, IRF1,* and *TNKS1BP1)* and a control (si*GFP*) using DharmaFECT3 according to the manufacturer's instructions (Dharmacon, http://www.dharmacon.com). Fibroblasts were transfected with 20 nM of siRNAs at a density of 2 × 10^5^ cells/well (six-well plate) in high serum (10% FBS) media. After 24 h, the treated fibroblasts were replated at a density of 6 × 10^3^ cells/well in four-well chamber slides and were allowed to recover in high serum for 48 h. The transfected cells were then transferred to low serum (0.1% FBS) media for 16 h. The transfection efficiency of each siRNA was verified by qRT-PCR (Figure S1). DNA synthesis was monitored by measuring the incorporation of the thymidine nucleotide analog BrdU (Sigma, http://www.sigmaaldrich.com) into DNA as previously described [11]. Briefly, cells were incubated with 10 μM BrdU in the media for 6 h; then washed with PBS, fixed, and stained with an anti-BrdU monoclonal antibody (Becton Dickinson, http://www.bd.com) and Alexa Fluor-conjugated goat anti-mouse antibody (Molecular Probes, http://www.invitrogen.com). The percentage of BrdU-positive cells among >200 DAPI-positive cells in four random fields was recorded. Propidium iodide staining of DNA content and FACS analysis were performed as described [38], with four replicate samples for each condition.

### Quantitative Reverse Transription-PCR.

Gene expression levels for genes targeted by the siRNAs were quantitated using RNA extracted from the transfected cells by Taqman quantitative one-step RT-PCR (Applied Biosystems, http://www.appliedbiosystems.com). Taqman probes to *SALL2*, *MXI1*, and *IRF1* were used. Assays were normalized to *GAPDH* levels, and relative abundance was calculated using a delta–delta threshold analysis as previously described [39]. The assay identification numbers for the Taqman probes are in the Accession Numbers list in the Supporting Information section of this paper.

## Supporting Information

Figure S1 Late Response Genes to SS and SD Show Symmetric Regulation Induction of cell cycle genes by SS and their coordinate repression by SD is shown. Our module map method identified two well-known cell cycle regulators—the joint motif of E2F:DP complexes as well as the motif for NFY—as enriched *cis*-regulatory sequences for these genes. Conversely, genes involved in sterol metabolism are repressed by SS (because of the presence of cholesterol in serum) and coordinately induced by SD. Our method also correctly identified the known key regulator, SREBP, by its enriched *cis*-regulatory motif.
(271 KB PDF)

Figure S2 Efficiency of siRNA Knockdown as Verified by Quantitative Reverse Transcription-PCR Relative mRNA levels (mean ± standard error) are shown. We compared the level of siRNA-mediated inhibition of gene expression in quiescent cells relative to the baseline level in asynchronously growing cells. The degrees of siRNA-mediated inhibition are 64% knockdown for *SALL2,* 100% knockdown for *MXI1,* and 48% knockdown for *IRF1.*
(30 KB PDF)

Table S1 SDERGs Listed are the SDERGs, which showed immediate induction within the first three hours after SD but demonstrated substantially less or delayed regulation by SS.
(12 KB PDF)

### Accession Numbers

The LocusLink (http://www.ncbi.nlm.nih.gov/entrez/query.fcgi?db=gene) and Unigene ID (http://www.sgn.cornell.edu/bulk/input.pl?mode=unigene) of genes discussed in this manuscript are listed in Table S1.

The National Center for Biotechnology (NCBI) Probe Database (http://www.ncbi.nlm.nih.gov/entrez/query.fcgi?db=probe) accession numbers for the Taqman probes discussed in this manuscript are *GAPDH,* Hs99999905_m1; *IRF1,* Hs00233698_m1; *MXI1,* Hs00365651_m1; and *SALL2,* Hs00826674_m1.

## Abbreviations

BrdU: 5-bromo-2′-deoxyuridine
FBS: fetal bovine serum
FDR: false discovery rate
SD: serum deprivation
SDERG: serum deprivation early response gene
si: silent interfering
siRNA: silent interfering RNA
SS: serum stimulation
