# Supplementary material for: A Transcriptional Program Mediating Entry into Cellular Quiescence
Source: PLoS Genet. 2007 Jun 8;3(6):e91. doi: 10.1371/journal.pgen.0030091 (PMC1904355; doi:10.1371/journal.pgen.0030091)
Supplement: Supplementary file 1 [file pgen.0030091.sg001.pdf]

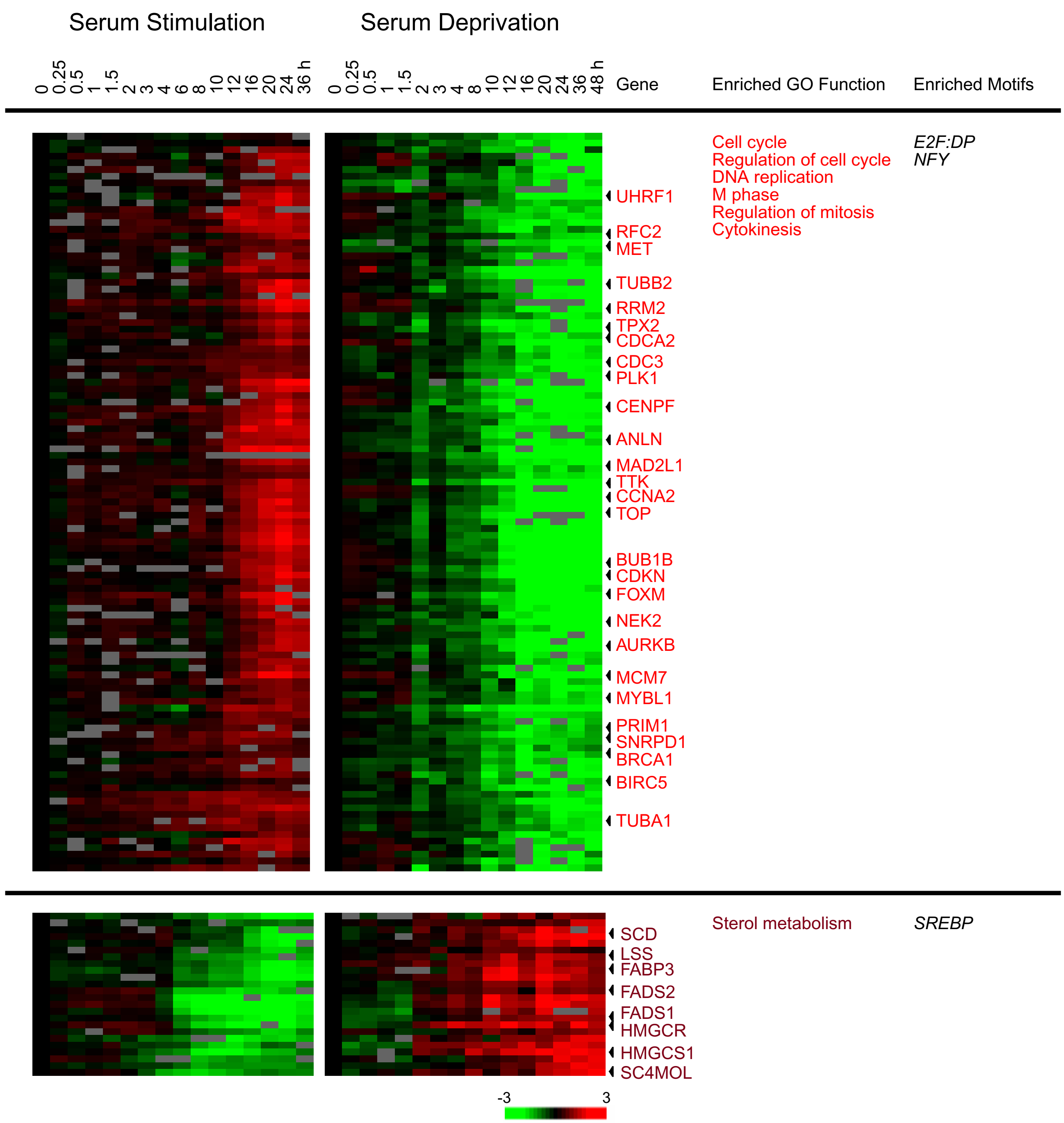
Serum Stimulation
Serum Deprivation
0 0.25 0.5 1 1.5 2 3 4 6 8 10 12 16 20 24 36 h
0 0.25 0.5 1 1.5 2 3 4 8 10 12 16 20 24 36 48 h
Gene
Enriched GO Function
Enriched Motifs
UHRF1
RFC2
MET
TUBB2
RRM2
TPX2
CDCA2
CDC3
PLK1
CENPF
ANLN
MAD2L1
TTK
CCNA2
TOP
BUB1B
CDKN
FOXM
NEK2
AURKB
MCM7
MYBL1
PRIM1
SNRPD1
BRCA1
BIRC5
TUBA1
Cell cycle
Regulation of cell cycle
DNA replication
M phase
Regulation of mitosis
Cytokinesis
E2F:DP
NFY
SCD
LSS
FABP3
FADS2
FADS1
HMGCR
HMGCS1
SC4MOL
Sterol metabolism
SREBP
-3
3
