# Supplementary material for: A Transcriptional Program Mediating Entry into Cellular Quiescence
Source: PLoS Genet. 2007 Jun 8;3(6):e91. doi: 10.1371/journal.pgen.0030091 (PMC1904355; doi:10.1371/journal.pgen.0030091)
Supplement: Supplementary file 2 [file pgen.0030091.sg002.pdf]

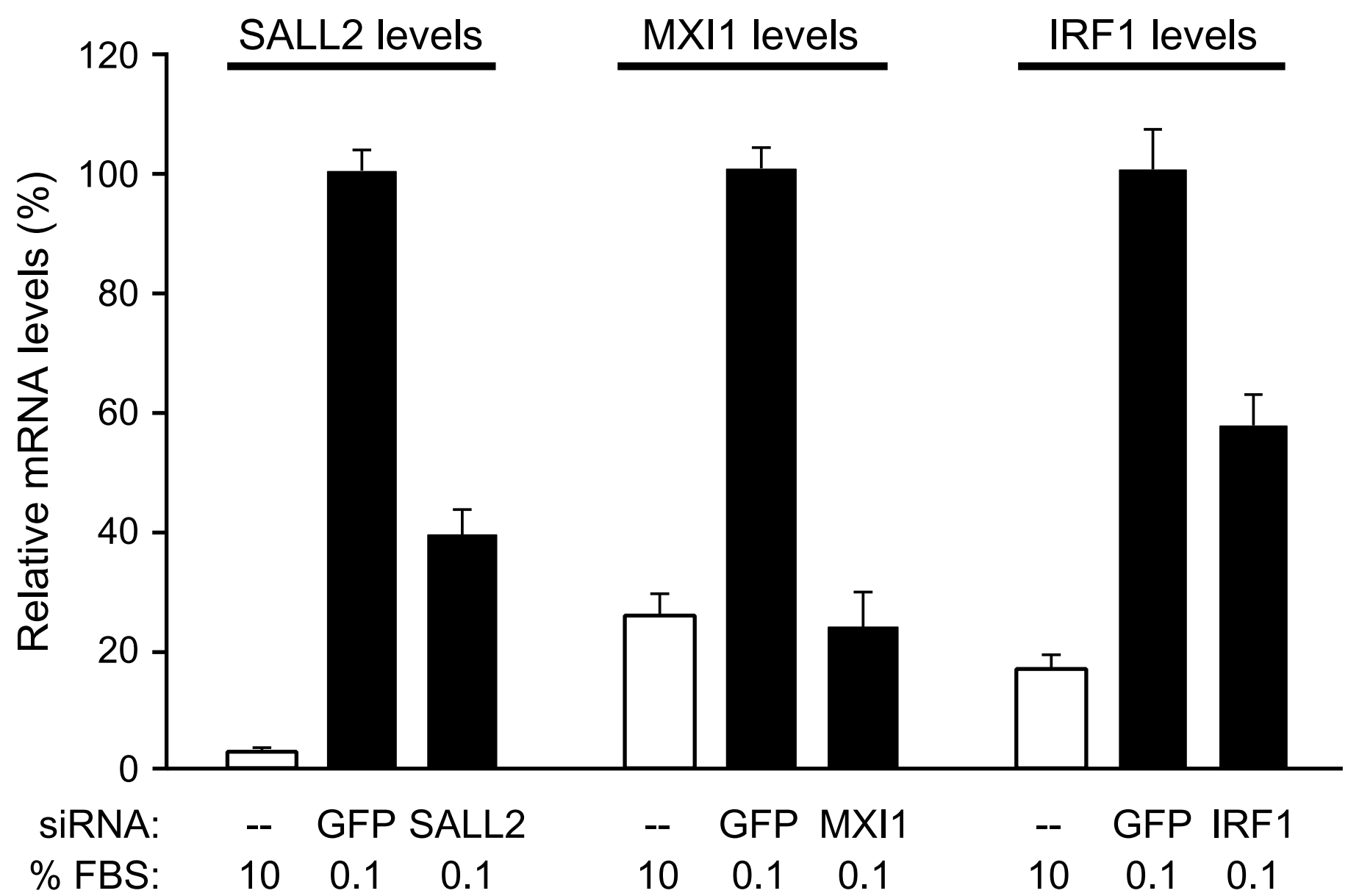
SALL2 levels
MXI1 levels
IRF1 levels
Relative mRNA levels (%)
0
20
40
60
80
100
120
siRNA:
--
GFP
SALL2
--
GFP
MXI1
--
GFP
IRF1
% FBS:
10
0.1
0.1
10
0.1
0.1
10
0.1
0.1
