# Supplementary material for: A Transcriptional Program Mediating Entry into Cellular Quiescence
Source: PLoS Genet. 2007 Jun 8;3(6):e91. doi: 10.1371/journal.pgen.0030091 (PMC1904355; doi:10.1371/journal.pgen.0030091)
Supplement: Supplementary file 3 [file pgen.0030091.st001.pdf]

Supplementary Table 1. Serum deprivation early response genes (SDERGs). Listed are the SDERGs, which showed immediate induction within the first three hours after serum deprivation but demonstrated substantially less or delayed regulation by serum stimulation.

Gene Symbol	LocusLink ID	UniGene Cluster ID
ACVRL1	94	Hs.591026
ANGPT1	284	Hs.369675
ATP1A1	476	Hs.371889
BDKRB2	624	Hs.525572
BNC1	646	Hs.459153
CDH2	1000	Hs.606106
COL1A1	1277	Hs.172928
COL3A1	1281	Hs.443625
COX7A1	1346	Hs.421621
CYP4B1	1580	Hs.436317
DOCK1	1793	Hs.159195
DPP4	1803	Hs.368912
EDG3	1903	Hs.585118
EWSR1	2130	Hs.374477
F10	2159	Hs.361463
FBLN2	2199	Hs.198862
FOXD1	2297	Hs.519385
FLT1	2321	Hs.507621
CXCL1	2919	Hs.789
HK2	3099	Hs.591588
TNC	3371	Hs.143250
IGF2R	3482	Hs.487062
IL1R1	3554	Hs.557403
IL6	3569	Hs.512234
IL6ST	3572	Hs.532082
IL11	3589	Hs.467304
ITGA2	3673	Hs.591770
ITPKB	3707	Hs.528087
KRT5	3852	Hs.433845
KRT34	3885	Hs.296942
L1CAM	3897	Hs.522818
LAMA2	3908	Hs.200841
LAMA4	3910	Hs.213861
LAMC1	3915	Hs.497039
LMO7	4008	Hs.207631
LNPEP	4012	Hs.527199
MAB21L1	4081	Hs.584776
MME	4311	Hs.307734
MTX1	4580	Hs.490874
MXI1	4601	Hs.501023

MYLK	4638	Hs.556600
NCF1	4687	Hs.520943
NOTCH3	4854	Hs.8546
PBX1	5087	Hs.493096
PRELP	5549	Hs.632481
PRKAR1B	5575	Hs.520851
PSG1	5669	Hs.466843
PTGS2	5743	Hs.196384
RGS4	5999	Hs.386726
SALL2	6297	Hs.416358
SATB1	6304	Hs.517717
SLC19A1	6573	Hs.84190
SLIT3	6586	Hs.604116
SLPI	6590	Hs.517070
STAT1	6772	Hs.470943
STC1	6781	Hs.25590
TBX3	6926	Hs.129895
TGFB1	7040	Hs.155218
THBS1	7057	Hs.164226
THBS3	7059	Hs.169875
TNFAIP3	7128	Hs.591338
TPR	7175	Hs.279640
TFPI2	7980	Hs.438231
FZD7	8324	Hs.173859
ADAM19	8728	Hs.483944
IQGAP1	8826	Hs.430551
LDB2	9079	Hs.23748
MSC	9242	Hs.442619
ISG15	9636	Hs.458485
C6orf32	9750	Hs.559459
ZFHX1B	9839	Hs.34871
LPPR4	9890	Hs.13245
HS3ST3B1	9953	Hs.48384
MXD4	10608	Hs.102402
PGRMC1	10857	Hs.90061
STON1	11037	Hs.44385
DKFZP686A01247	22998	Hs.335163
TBC1D2B	23102	Hs.567426
SULF1	23213	Hs.409602
EHBP1	23301	Hs.271667
ZNF629	23361	Hs.301094
LMOD1	25802	Hs.519075
MIZF	25988	Hs.504091
WSB1	26118	Hs.446017
GREM1	26585	Hs.40098
NBEA	26960	Hs.491172
TRA2A	29896	Hs.445652
TRHDE	29953	Hs.199814

LMCD1	29995	Hs.475353
COL5A3	50509	Hs.235368
ANKHD1	54882	Hs.434219
HCFC1R1	54985	Hs.423103
C9orf46	55848	Hs.584242
BEX1	55859	Hs.334370
C14orf132	56967	Hs.6434
KIAA1219	57148	Hs.436705
ZNFX1	57169	Hs.371794
ABHD6	57406	Hs.476454
BIRC6	57448	Hs.150107
MARCH4	57574	Hs.170388
MARK4	57787	Hs.34314
NTN4	59277	Hs.201034
ELTD1	64123	Hs.132314
ZFP106	64397	Hs.511143
TNS3	64759	Hs.520814
GNPTAB	79158	Hs.46850
ZYG11B	79699	Hs.476280
CYBRD1	79901	Hs.221941
FLJ20920	80221	Hs.288959
SPRY4	81848	Hs.323308
C8orf13	83648	Hs.124299
PARP9	83666	Hs.518200
LRRC8C	84230	Hs.412836
C15orf48	84419	Hs.112242
FLJ14834	84935	Hs.616329
KIAA1754	85450	Hs.523252
TNKS1BP1	85456	Hs.530730
DPP9	91039	Hs.515081
FBXO32	114907	Hs.403933
GSTO2	119391	Hs.203634
C18orf17	125488	Hs.128576
HSPB6	126393	Hs.534538
FLJ37440	129804	Hs.437696
GPR175	131601	Hs.6418
SAMD9L	219285	Hs.489118
OAF	220323	Hs.445081
LOC220686	220686	Hs.593045
SCUBE3	222663	Hs.12923
MKX	283078	Hs.128193
TMEM119	338773	Hs.449718
FAM111B	374393	Hs.186579
MALAT1	378938	Hs.556259
VGLL3	389136	Hs.606507
LOC653160	653160	Hs.632392
